# Vulnerability- and Diversity-Aware Anonymization of Personally Identifiable Information for Improving User Privacy and Utility of Publishing Data

**DOI:** 10.3390/s17051059

**Published:** 2017-05-08

**Authors:** Abdul Majeed, Farman Ullah, Sungchang Lee

**Affiliations:** 1 School of Information and Electronics Engineering, Korea Aerospace University, Deogyang-gu, Goyang-si, Gyeonggi-do 412-791, Korea; abdulmajid09398@kau.kr; 2 Innovergence Lab (NGN Lab), Korea Aerospace University, Hanggongdaehang-ro, Deokyang-gu, Goyang-si, Gyeonggi-do 412-791, Korea; farmankttk@ciit-attock.edu.pk

**Keywords:** personally identifiable information, identity vulnerability, diversity, adaptive generalization, privacy, utility

## Abstract

Personally identifiable information (PII) affects individual privacy because PII combinations may yield unique identifications in published data. User PII such as age, race, gender, and zip code contain private information that may assist an adversary in determining the user to whom such information relates. Each item of user PII reveals identity differently, and some types of PII are highly identity vulnerable. More vulnerable types of PII enable unique identification more easily, and their presence in published data increases privacy risks. Existing privacy models treat all types of PII equally from an identity revelation point of view, and they mainly focus on hiding user PII in a crowd of other users. Ignoring the identity vulnerability of each type of PII during anonymization is not an effective method of protecting user privacy in a fine-grained manner. This paper proposes a new anonymization scheme that considers the identity vulnerability of PII to effectively protect user privacy. Data generalization is performed adaptively based on the identity vulnerability of PII as well as diversity to anonymize data. This adaptive generalization effectively enables anonymous data, which protects user identity and private information disclosures while maximizing the utility of data for performing analyses and building classification models. Additionally, the proposed scheme has low computational overheads. The simulation results show the effectiveness of the scheme and verify the aforementioned claims.

## 1. Introduction

Most organizations collect relevant customer data to improve service quality. The excessive amount of collected data often contains information on customer demographics, finances, interests, activities, and medical status. Research has shown that access to this data can aid organizations in many ways. For instance, it allows them to provide customer analyses, achieve strategic goals, create effective marketing strategies, and improve overall business performance. However, organizations are not willing to publish person-specific data because personally identifiable information (PII) often leads to unique individual identification. A survey [1] reported that:

About 87% of the population in the United States is likely to be identified based only on 5-digit zip code, gender, date of birth. About 50% of the U.S. population are likely to be uniquely identified by only place, gender, date of birth. Moreover, even at the county level, county, gender, date of birth are likely to identify about 18% of the U.S. population.

User PII such as age, gender, place, zip code, and race are called quasi-identifiers (QIs). Each QI affects user privacy differently, and some QIs, like zip codes and places, are highly identity vulnerable. QIs with high identity vulnerability enable easier unique identification. The presence of identity-vulnerable QIs in published data increases privacy risks for sensitive attributes (SAs) such as diseases or salary information disclosure. Therefore, it is necessary to anonymize user data in a fine-grained manner before publishing it, and in many cases, this is achieved by employing anonymization. This is a practical approach to achieve privacy-preserving data publishing (PPDP) [2]. The strategy mainly uses generalization and suppression techniques to anonymize the person-specific data.

Mostly the existing anonymization schemes do not provide thorough insights into privacy, particularly regarding the identity vulnerability of QIs and the diversity of SA-based adaptive data generalization. Current privacy schemes mainly focus on hiding individual QIs in the crowd of other users and treat all QIs equally from an identity revelation point of view. However, in many real-world cases, each QI reveals identity differently (e.g., a zip code represents a local region more accurately than a country or place). Therefore, it is mandatory to consider the identity vulnerability of each QI and the diversity of SAs to effectively protect user privacy. Recently, information surges and advances in machine learning tools have created unexpected privacy concerns. These tools are very good at finding the private information of individuals from large datasets using relevant QIs with high probabilities [3,4,5], which cannot be eliminated by applying the privacy models discussed so far. The power of machine learning tools is increasing aggressively, and it is expected to double in the next few years [6]. Therefore, considering the limitations of the existing work, and the continually growing power of machine learning tools, PPDP has received global attention over the past few decades. There is an emerging need to develop methods that use the capabilities of these tools to protect user privacy.

Generally, there are two settings for privacy preserving data publishing: interactive and non-interactive. In non-interactive settings, the data owner (e.g., hospitals, insurance companies and/or service providers), a trusted party, publishes the complete dataset in perturbed form after applying some operation to original data and removing directly identifiable information (e.g., name). However, in interactive settings the data owner, a trusted party, does not publish the whole dataset in perturbed form like in the non-interactive setting. It provides an interface to the users through which they may pose different queries about the data and get (possibly noisy) answers. Each of the privacy settings introduces various privacy concerns pertaining to the users sensitive and demographics data in question. Hence, one approach cannot replace the other, and they each have a place alongside the other.

Differential privacy (DP) [7] has emerged as a state-of-the-art method and one of the most promising privacy models for releasing person-specific data in interactive settings. DP offers strong privacy guarantees and it has been developed in the sequence of papers [8,9,10,11]. It provides the desired level of privacy and based on the fact that presence or absence of any individuals in the database does not significantly influence the results of the analyses on the dataset. However, enforcement of these strict guarantees in real-world applications may not fulfill either strong privacy guarantees or desired data utility requirements. The users can execute different statistical queries and can get the noisy and aggregate answers from the differentially private systems. Currently, various approaches have been proposed to extend the DP concept for data mining. In this regard, a comprehensive study was given by the authors of [12], and they verified the fact that DP is typically applicable for privacy preserving data mining. The authors of [13] study, discuss, and formalize an alternative to the standard DP model as individual differential privacy model that introduces less amount of noise to the query results for improving utility/accuracy. Sarathy et al. [14] reported a method for adding noise in response to user queries to protect privacy. After the query function is computed from the original data set, Laplace-distributed random noise is added to the query result. Generally, the magnitude of the noise depends on the sensitivity of the query function and desired privacy budget for that specific query. The authors concluded that noise variance should vary with the subsequent queries to deliver promised level of privacy at the expense of utility. There are many differentially private systems, such as privacy-integrated queries (PINQ) [15], Airavat [16] and Gupt [17]. These systems maintain each query budget and mediate accesses to the underlying data in a differentially-private manner. These systems maintain various levels of privacy for different user’s applications while ensuring the desired level of privacy and utility.

In this work, we focus on the non-interactive setting of person-specific data publishing, and extend the *k*-anonymity [18] concept and its ramifications as a very popular privacy model within the research community. The *k*-anonymity [18] is a well-known PPDP technique that protects user privacy by introducing *k*-users with the same QIs in each equivalence class. Thus, the probability of re-identifying someone in anonymized data becomes at least 1/k. This helps to protect against a linking attack with external data sources by ensuring *k* or more identical tuples in each equivalence class. However, user privacy is not guaranteed when an adversary has strong background knowledge or when the SAs in an equivalence class are not diverse. Many attempts have been made to enhance the protection level of *k*-anonymity. Three different PPDP models—*ℓ*-diversity [19], (α,k)-anonymity [20], and *t*-closeness [21]—have been proposed to address *k*-anonymity limitations. They impose more protective requirements, consider diversity, and ensure the equal proportion of SAs in each class to overcome private information disclosure. However, creating a feasible *ℓ*-diverse dataset is very challenging when the data is highly imbalanced (e.g., if the SA distribution is uneven). In such datasets, maintaining user privacy through *ℓ*-diversity or *t*-closeness is not possible in practice, and the current schemes do not guarantee individual privacy. To overcome the aforementioned limitations, this study proposes a new anonymization scheme that reduces the privacy risks while improving the anonymous data utility in person-specific data publishing. When the data is anonymized, data generalization is performed adaptively based on the identity vulnerability of the QIs as well as the diversity of SAs in each class.

The remainder of this paper is organized as follows: Section 2 explains the background and related work regarding well-known PPDP models. Section 3 presents the conceptual overview of proposed anonymization scheme and outlines its principal steps. Section 4 provides a brief overview about determining the identity vulnerability of the QIs using random forest, and Section 5 describes the adaptive generalization algorithm. Section 6 discusses the simulation and results. Finally, conclusions are offered in Section 7.

## 2. Background and Related Work

**Figure 1:**
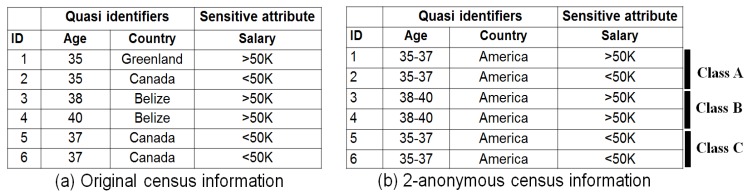
*k*-anonymity privacy model overview.

Privacy protection has remained a concern for researchers and experts because recent advances in information technology have threatened user privacy. Information surges have made the retrieval of QIs along with the SAs of individuals a part of day-to-day life. PPDP provides promising methods for publishing useful information while preserving data privacy [22]. In PPDP, explicit identifiers (e.g., name) are removed, and QIs are generalized or suppressed to protect the SAs of individuals from disclosure. Researchers require sensitive attributes, so they are mostly retained unchanged. In many circumstances, *k*-anonymity [18] provides sufficient protection because of its conceptual simplicity. It is a well-known PPDP model, and due to algorithmic advances in creating *k*-anonymous datasets [23,24,25,26], it has become a benchmark. Figure 1a shows selected census information for a group individuals. In this example, attributes are divided into two groups: QIs and SA. Figure 1b shows a 2-anonymous table derived from the table in Figure 1a.

Each class has two tuples with identical QIs. However, two simple attacks—homogeneity and background knowledge on *k*-anonymous datasets—allow an adversary to infer the SA of an individual with ease. Consider the two simplest attacks on anonymous data in Figure 1b produced by the *k*-anonymity model. Juan and Tana are two Belizean friends, and one day they have dinner together. Juan tells his friend, Tana, that he took part in a census conducted by the National Institute of Statistics (NIS) last week. After knowing this information, Tana sets out to discover Juan’s salary. She discovers the 2-anonymous table of current census participants published by the NIS (Figure 1b). She knows that one of the records in this table must contain Juan’s salary information. Juan is Tana’s neighbor also, so she knows that Juan is a 40-year-old resident of Belize. She discovers that Juan must be either 3 or 4 in Figure 1b. Now, all participants have the same finance information (e.g., salary is greater than 50,000 dollars). Therefore, Tana concludes that Juan’s salary is greater than 50,000 dollars.Tana has a friend named Albert from Canada, who also participated in the same census, and whose financial status also appears in the table shown in Figure 1b. Tana knows that Albert is a 35-year old who lives in Canada permanently. Based on this information, Tana determines that Albert is represented by either the first, second, fifth, or sixth row in Figure 1b. Without any other information, she is not sure whether Albert has a salary higher or lower than 50,000 dollars. However, it is well known that majority of Canadian adults have salaries less than 50,000 dollars. Therefore, Tana concludes with near certainty (e.g., 75%) that Albert’s salary is 50 K. Thus, it is evident that *k*-anonymity can create groups that are prone to leaking private information.

A stronger definition of privacy that resolves the aforementioned limitations of *k*-anonymity is *ℓ*- diversity [19]. The main idea behind a *ℓ*-diverse dataset is that the values of the SAs in each class should be well represented (e.g., the same as *ℓ*). Class *A* in Figure 1b has a diversity of 2, while classes *B* and *C* have no diversity at all. Achieving *ℓ*-diversity becomes very challenging when the SA distribution is uneven, such as with these two values: salary > 50 K (1%) and < 50 K (99%). In many other cases as well, *ℓ*-diversity is hard to achieve, such as for classes *B* and *C* in Figure 1b, which contain only salary values higher or lower than 50 K. To overcome these issues, *t*-closeness [21] was proposed. In the *t*-closeness model, the distribution of SAs in any QI group and the overall table should be no more than a certain threshold. However, *t*-closeness does not protect from attribute disclosure and is complex in nature. Another significant contribution regarding the implications from the QIs to SAs made by using a local recording-based algorithm to enhance the protection level of *k*-anonymity is (α,k)-anonymity [20]. The advantage of the (α,k)-anonymity model is that it can be extended to more general cases (e.g., two or more SAs).

A growing body of literature has examined the anonymous data utility in terms of classification models and their accuracy. A most recent review of the literature on retaining maximum utility in anonymous data from classification accuracy perspective was offered by [27]. They make use of DP for anonymizing and publishing data that shows considerable improvements in accuracy using decision trees. Apart from this aspect, currently much work on the potentials of data mining has been carried out to mine different perspectives and summaries from data and convert them into useful information. In this regard, a comprehensive study was given by the authors of [28]; the proposed technique has shown considerable improvements in privacy and utility based on the relations among different attributes in the dataset. The authors make use of the of entropy and information gain to find the distributions of data to protect privacy. The proposed study offers the potential for finding several relations based on data background and area of application. Another independent study to publish useful dataset to satisfy certain research needs, e.g., classification was proposed by the authors of [29] after adding some noise in the released data. The authors of [30] study proposed a comprehensive solution for protecting individual privacy using a rule-based privacy model that protects against explicit identity and private information disclosures while releasing person-specific data. It has been suggested that the proposed model recursively generalizes data with low information loss, and ensures promising scalability using sampling and a combination of top-down and bottom-up generalization heuristics. A general-purpose strategy to improve structured data classification accuracy by enriching data with semantics-based knowledge obtained by a multiple-taxonomy built over data items was provided by the authors of [31], and this seems to be a reliable solution for producing more useful datasets for analysis or building classification models.

A related review of the literature on utility-aware anonymization was offered by [32,33]. They draw attention to attribute-level anonymization via retaining the original values of some useful QIs to the greatest extent possible to enhance the utility of the anonymous data. The main weakness of their studies is that they make no attempt to consider the identity vulnerability of the QIs in terms of privacy. Recently, classification models for anonymous data have gained popularity. However, this requires the identification and modeling of privacy threats when releasing data [34]. Likewise, knowledge of the dependencies between QIs and SAs (e.g., male patients are less likely to have breast cancer) [35,36], the underlying anonymization methods [37], and the combination of QI values [38], may affect privacy. However, such types of threats have been overlooked in previous work. Data mining reveals knowledge patterns that apply to many people in the data [39], and the existing privacy models often ignore these receptive patterns. An independent study in 2008 [40] discussed *k*-anonymity in data mining and seemed to be reliable. Anonymity is achieved by extending the general *k*-anonymity model with the data mining model, and the proposed algorithm provides reasonable privacy in data mining.

A number of studies have explored a closely-related method used in data publishing for improved classification utility and privacy, such as InfoGain Mondrian [41], top down refinement (TDR) [42], information-based anonymization for classification given *k* (IACk) [43] and *k*-anonymity of classification trees using suppression (kACTUS) [44]. Interestingly they produced the same 2-anonymous table as the one shown in Figure 1a with partial or full suppression of age or country attributes. Mondrian [41] is a multidimensional method that partitions the data into disjointed rectangular regions according to QI values. Each rectangular region contains at least *k*-data points to facilitate value generalization. A serious weakness of the Mondrian method is its unnecessary suppression, which fully hides the QI values. kACTUS [44] and TDR [42] will produce the same results by employing the information gain versus anonymity and classification trees, respectively, during anonymization. These approaches are not well suited to privacy because consideration is not given to the probability that each attribute will reveal an individual’s identity. The IACk [43] algorithm reasonably improves the classification accuracy by considering mutual information when selecting the generalization level. However, SA diversity is not considered, which makes private information disclosure obvious. An even greater source of concern is the need to control the suppression of QIs having less identity vulnerability as much as possible to facilitate utility [45]. Suppression is a convenient solution to preserve privacy, and most of the machine learning tools can handle suppressed values as missing values. However, in large datasets, full column suppression of less vulnerable QI values potentially hurts utility. Apart from this utility aspect, most of the existing privacy algorithms over-fit anonymized data to QIs, and this over-generalization results in high information losses [46]. Therefore, anonymous data produced by the existing schemes have less utility. Accordingly, identity cannot be effectively protected and private information disclosure cannot be adequately prevented in a fine-grained manner. The contributions of this research in the field of PPDP can be summarized as follows: (1) it can be used for the anonymization of any dataset, balanced or imbalanced; (2) it uses random forest, a machine learning method, to determine the identity vulnerability values of QIs to reduce the unique identifications caused by the highly identity vulnerable QIs; (3) the Simpson index is used to determine the diversity of classes. A threshold value for the comparison of each class diversity is provided, and considerable attention is paid to low diverse classes to overcome privacy breaches; and (4) we proposed an adaptive generalization scheme for anonymizing data considering the identity vulnerability of QIs as well as the diversity of SAs in equivalence classes to improve both user privacy and utility as compared to existing PPDP algorithms.

## 3. The Proposed Anonymization Scheme

**Figure 2:**
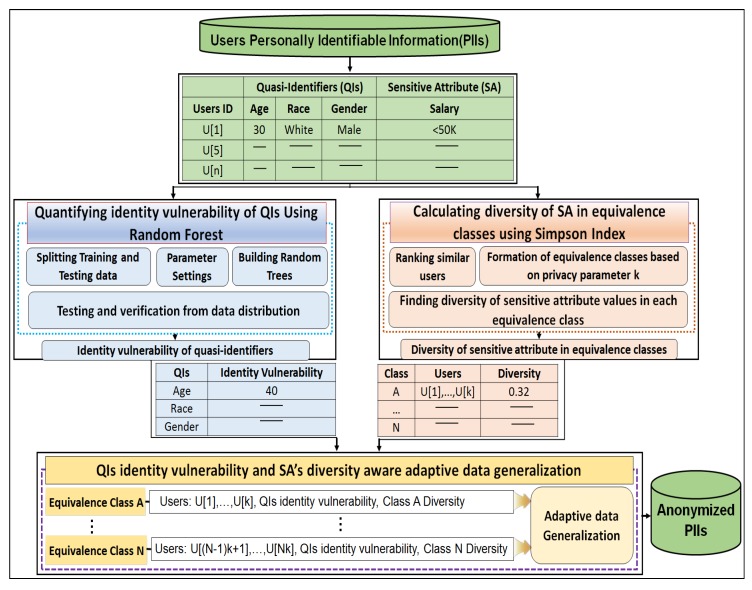
Conceptual overview of the proposed anonymization scheme.

The QI identity vulnerability- and SA diversity-aware anonymization scheme is necessary to account for the privacy issues stemming from the highly identity vulnerable QIs and low diverse classes. This scheme not only protects privacy, it augments utility by controlling over-generalization of less identity-vulnerable QIs in diverse classes. This section presents the conceptual overview of the proposed anonymization scheme and outlines its procedural steps. Figure 2 shows the conceptual overview of our proposed anonymization scheme.

To anonymize any person-specific data containing QIs and SAs, the following five principal concepts are introduced: (1) the concept of QI’s identity vulnerability (IV); (2) highest similarity user ranking based on QIs values; (3) the formation of equivalence classes ($C_{i}$) using privacy parameter *k*; (4) calculating the diversity (*D*) and evenness (*E*) of the equivalence classes; and (5) adaptive data generalization considering both the identity vulnerability of the QIs and diversity of the SA in equivalence classes. This approach is chosen to enhance user privacy in any dataset and to reduce privacy breaches caused by the highly identity-vulnerable QIs and low diverse classes. The identity vulnerability of each QI is determined using random forest, and diversity is determined using the Simpson index. Both measures are considered during data anonymization. Brief details of the principal components with equations and procedures are as follows.

### 3.1. Determining the Identity Vulnerability Values of QIs

To provide fine-grained privacy, the identity vulnerability (IV) of each QI is determined before data anonymization. Random forest (RF) [47], a machine learning method, is used to determine the identity vulnerability of the QIs. This provides a formal solution to treat each QI according to its identity revelation ability. A detailed procedure for determining the identity vulnerability of the QIs using RF is explained in Section 4.

### 3.2. Highly Similar Users Ranking and Formation of Equivalence Classes

Based on QI values, similar users are ranked, this is done by means of cosine similarity given as:

(1) $$Sim\left(U1,V1\right)=\frac{\sum_{n=1}^{N}U1_{\left(n\right)}\times V1_{\left(n\right)}}{\sqrt{\sum_{n=1}^{N}{\left(U1_{\left(n\right)}\right)}^{2}}\times \sqrt{\sum_{n=1}^{N}{\left(V1_{\left(n\right)}\right)}^{2}}}$$

where U1 and V1 are two different users having QIs, $U1_{1},V1_{1},U1_{2},V1_{2},\cdots ,U1_{n},V1_{n}$. The resultant matrix contains highly similar users. Later, the user matrix is partitioned into different equivalence classes ($C_{1},\cdots ,C_{N}$) based on the privacy parameter (*k*). The value of *k* is selected by the data owner, and it can be any whole number. If highly similar users are *N*, the number of equivalence classes ($C_{i}$) can be obtained using Equation (2).

(2) $$C_{i}=\frac{N}{k}$$

### 3.3. Calculate and Compare Diversity and Evenness of Equivalence Classes

Ensuring sufficient diversity in each equivalence class ($C_{i}$) has a range of advantages, including better privacy preservation. To calculate the diversity (*D*) and evenness (*E*) of each equivalence class, $C_{i}$, the Simpson index is employed [48]. It is a very simple and reliable measure. The following procedure is used to calculate the *D* and *E* values. The complete procedure of calculating diversity and evenness of each equivalence class is given below.

1. Calculate the proportion ($p_{i}$) of each SA’s category in an equivalence class using (Equation (3)).
  (3) $$p_{i}=\frac{n_{i}}{k}$$
2. Sum and square the individual proportions ($p_{1},p_{2},p_{3},\cdots ,p_{n}$) of each SA’s category in an equivalence class.
  (4) $$\sum_{n=1}^{n}P_{i}^{2}={\left(p_{1}\right)}^{2}+{\left(p_{2}\right)}^{2}+{\left(p_{3}\right)}^{2}+,.....,+{\left(p_{n}\right)}^{2}$$
3. Reciprocate the value obtained from Equation (4). The result is diversity denoted with *D*.
  (5) $$D=1/\sum_{n=1}^{n}P_{i}^{2}$$
4. To find *E*, divide *D* by the total number of unique SA categories (*n*) in an equivalence class.
  (6) E=D/n

Later, we compare the *D* and *E* values with a defined threshold ($T_{d}=1.75$ for *D*, $T_{e}=0.75$ for *E*) for each class to preserve users privacy in better way. These values can be adjusted according to the protection level and objectives of data publishing.

### 3.4. Adaptive Data Generalization

Adaptive generalization selects the generalization level for each QI based on its identity vulnerability as well as the corresponding class’s diversity. Great care must be taken during the anonymization of less diverse classes to protect privacy. After the appropriate generalization level selection, the original values are replaced with generalized values to anonymize the data. A detailed discussion about the adaptive data generalization along with algorithm and its flow-chart is provided in Section 5.

## 4. Finding the Identity Vulnerability of Quasi Identifiers

This section presents the proposed mechanism by which the identity vulnerability of the QIs is determined using RF [47]. RF is a machine learning method, and we used it to quantify the identity vulnerability values of each QI and to identify vulnerable QIs that explicitly reveal users identities. Determining the identity vulnerability of QIs enables a formal solution to be derived that can effectively protect individual privacy in a fine-grained manner. Without considering the identity vulnerability of the QIs, it is not clear whether the anonymous tuple (e.g., full attribute set representing an individual) produced by any anonymization scheme to tackle user privacy is suitable for use in many real-world cases or not. Knowledge of the identity vulnerability of each QI to a fine level of granularity leads to better privacy preservation and fewer private information disclosures. It is worth looking at RF in greater detail, as well as at the procedure of determining the identity vulnerability of QIs.

**Figure 3:**
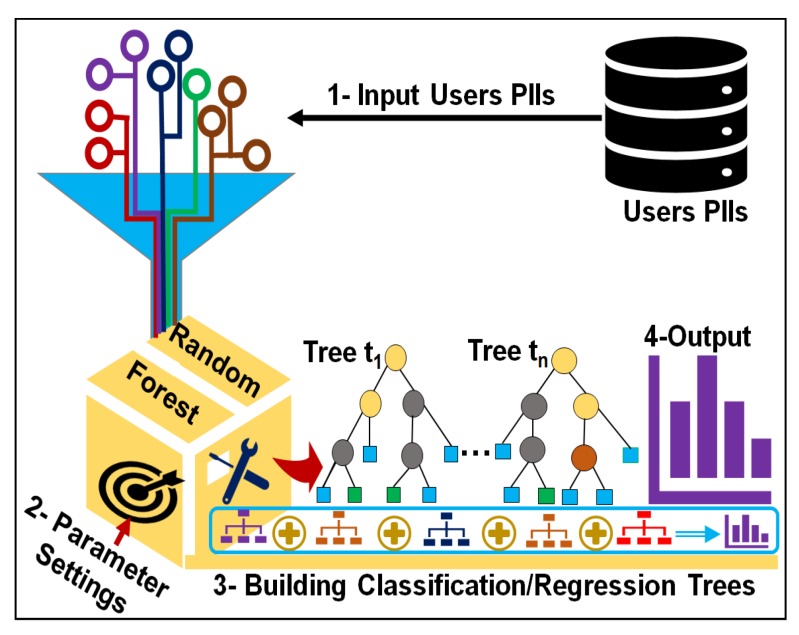
Steps of determining the identity vulnerability of QIs.

RF is a versatile machine learning method and a special type of ensemble learning technique. It was selected because among currently available algorithms, it is highly accurate. It considers the interaction between attributes and gives high accuracy values. It has been used extensively to build classification and regression models. It produces an ensemble of classification and regression trees (CARTs) using bagged samples of training data [49] as shown in Figure 3. By employing the bagging concept, each tree selects only a small set of QIs to split the tree node, which enables the algorithm to quickly build classifiers for highly dimensional data. RF is potentially attractive for handling large amounts of data and variables. It is the best among existing methods in dealing with missing values and outliers that exist in the training data, and gives better accuracy values [50]. It provides more than six different numerical measures regarding the observations (number of records used as input), including classification accuracy, misclassification rate, precision, recall, F-statistic, and variable importance. However, the most important measure among them is variable importance because it assists in categorizing QIs according to their identity revelation ability. A QI having high importance reveals an identity more easily than other QIs. RF has only two parameters—the number of trees (ntree) and the number of QIs required at the node split (mtry)—so it is very easy to tune them. In standard terms, these are called parameter settings, which allow data practitioners to modify these two parameter values subject to their data and purpose. A general procedure to quantify the identity vulnerability of QIs using RF is presented visually in Figure 3, and the corresponding algorithm pseudo code is provided in Algorithm 1. There are three major steps used to obtain the desired values of the identity vulnerability of the QIs—the data input, parameter settings, and building of the CARTs. The complete pseudo-code used to quantify the identity vulnerability of the QIs using RF is presented in Algorithm 1.

In Algorithm 1, a user dataset containing *N* number of records, and a set of QIs (*P*), a number of trees (*B*), and a small subset of QIs (*m*) used to split the tree node is provided as input. The identity vulnerability (IV) values of the QIs are obtained as output. RF builds an ensemble of CARTs and calculates the out-of-bag (OOB) error (Lines 1–5). We partition the original dataset into two parts during experiments, training data and testing data. So, two-thirds in Line 4 is the amount of training data (e.g., the data on which the algorithms were tested) containing 30,162 tuples out of 45,222 tuples and the remaining one-third of the data in Line 5 containing 15,060 was used for validation and testing purposes.

**Algorithm 1:** Finding identity vulnerability values of the QIs
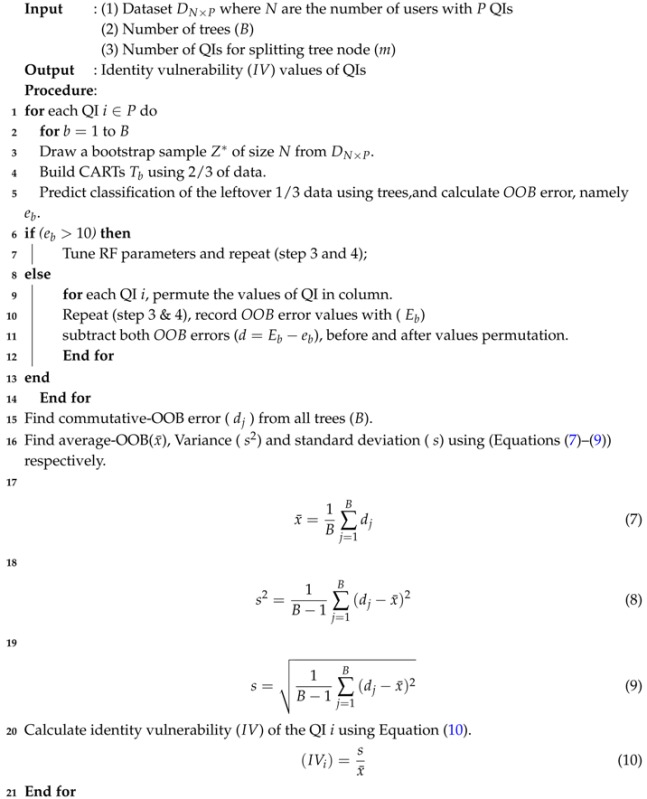


Integer 10 in line 6 represents the desired value of OOB error called misclassification produced by the random forest while building random trees. The desired values of accuracy should be higher than 90% to correctly measure the vulnerability values of quasi-identifiers. Therefore, we rigorously compare OOB error with integer 10 to keep the accuracy values higher. However, accuracy values can be adjusted according to the protection level and objectives of data publishing. If the OOB is high, then a parameter setting is performed to achieve the appropriate accuracy (Lines 6 and 7). In contrast, if the OOB falls within the acceptable range, then the values of each QI are shuffled in a column, and its impact on the OOB is observed (Lines 9–12). The same process is repeated for all QIs in a dataset. After getting the OOB values for all of the QIs, the mean ($\bar{x}$), variance ($s^{2}$) and standard deviation (*s*) are calculated using Equations (7)–(9). Subsequently, the identity vulnerability (IV) of each QI is calculated using Equation (10).

## 5. Determining the Best Generalization Level

Apart from the full suppression of all QIs in a tuple, complete safeguarding against the background knowledge and linking attacks as explained in Section 2, is very difficult. However, adaptive data generalization can play a significant role in guarding against these attacks to an acceptable level. This section presents the proposed adaptive generalization scheme appropriate generalization level selection from taxonomies based on the identity vulnerability of QIs and diversity of SA to effectively protect user privacy. After determining the identity vulnerability of the QIs, ranking highly similar users, and forming equivalence classes using *k*, it is now possible to calculate and compare the diversity of classes and determine the generalization level to anonymize QIs. When anonymizing the data, data generalization is performed adaptively based on the identity vulnerability of the QIs as well as the diversity. Apart from class diversity (*D*), evenness (*E*) is also calculated to determine the relative abundance of the SA values. Both of these measures are calculated using the Simpson index. The *D* and *E* values are calculated using Equations (3)–(6) (this procedure is explained in Section 3). Values with defined thresholds ($T_{d}$ and $T_{e}$) are subsequently compared. Because certain data statistics such as the identity vulnerability of the QIs and the diversity of classes are determined in advance, adaptive generalization (i.e., taxonomy positions are established) can be performed. For less diverse user classes, great care is taken in the corresponding QI group. The proposed adaptive generalization method, which couples the identity vulnerability of the QIs with the diversity of the SAs, ultimately provides considerable protection against various attacks. The adaptive generalization facilitates superior data anonymity, thereby protecting identities and preventing private information disclosures. At the same time, the data utility is also preserved. The technique shows clear improvements over existing methods of privacy protection that determine the generalization level based on mutual information or heuristics.

### 5.1. Higher and Lower Level Generalization

**Figure 4:**
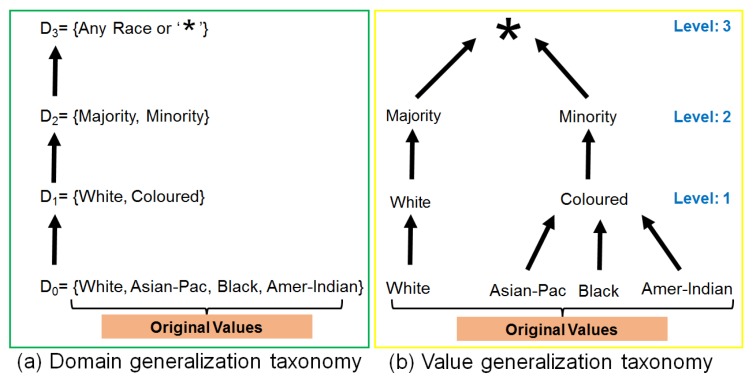
Example of domain and value generalization taxonomies of the race attribute.

Data generalization to anonymize QI values is based on either domain generalization hierarchy/taxonomy or value generalization hierarchy/taxonomy. A domain generalization taxonomy is defined to be a set of domains that is totally ordered by the relationship from lower limit to higher limit in numeric data, and all possible values ordered semantically or logically in case of alphabetic data. We can consider the taxonomy as a chain of nodes, and if there is an edge from node $D_{a}$ (bottom node) to another node $D_{b}$ (top node) of the same branch in generalization taxonomy, it means that $D_{b}$ is the direct generalization of $D_{a}$. Similarly, if $Dom_{x}$ is a set of domains in a domain generalization taxonomy of a QI from QIs set, $Q=\{q_{1},q_{2},q_{3},\cdots ,q_{n}\}$. For every $D_{a},D_{b}$ and $D_{c}$ belonging to $Dom_{x}$ if $D_{a}<D_{b}$ and $D_{b}<D_{c}$, then $D_{a}<D_{c}$. In this case, domain $D_{c}$ is an implied generalization of $D_{a}$. Figure 4 shows an example of generalization taxonomies of race attribute. Domain generalization taxonomy of race is given in Figure 4a. Meanwhile, the value generalization functions associated with the domain generalization taxonomy induces a corresponding value-level taxonomy based on the actual QI values. In value generalization taxonomy, edges are denoted by *r*, i.e., direct value generalization, and paths are denoted by $r^{+}$, i.e., implied value generalization. Figure 4b shows a value generalization taxonomy with each value in the race, e.g., colored = *r* (black) and * belongs $r^{+}$ (black). For all QIs, $Q=\{q_{1},q_{2},q_{3},\cdots ,q_{n}\}$ consisting of multiple values, each with its own domain. The domain generalization hierarchies of the individual attributes can be combined to form generalization lattices and combine taxonomies to be used for anonymizing QIs values. In Algorithm 2, we mainly refer to direct generalization (generalization at lower levels close to original values, e.g., level 1 or 2 in Figure 4) as lower level generalization, and implied generalization (generalization at higher levels close to the root node, e.g., level 2 or 3 in Figure 4) as higher level generalization. When the diversity of sensitive attributes is less than desired threshold then higher level generalization is preferred to limit privacy breaches. Meanwhile, lower level generalization is performed to preserve anonymous data utility for performing analysis or building different classification models.

### 5.2. Adaptive Data Generalization Algorithm

**Figure 5:**
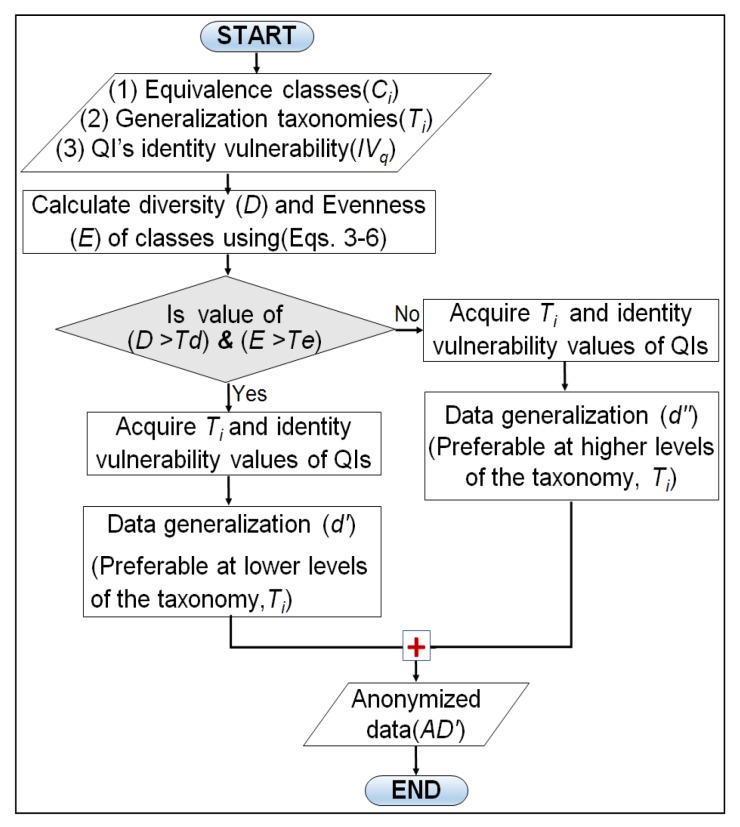
Adaptive generalization algorithm’s flow chart.

In this section, we present proposed adaptive generalization algorithm and its working with the help of flowchart and pseudo-code. The proposed algorithm performs two major steps to produce anonymous data from the original data: finding *D* and *E* values and their analyses, and adaptive data generalization. The *D* and *E* analyses steps involve comparing each class diversity and evenness with their defined thresholds ($T_{d}$ and $T_{e}$). However, the adaptive data generalization step selects the most appropriate generalization level to anonymize QI values. Figure 4 shows three different levels to anonymize race values given in the dataset. As we climb up in the taxonomy tree, the distance from the original values increase and information becomes less specific. Data generalization near the root of the tree offers strong privacy guarantees but utility will be very low. In either case, data generalization at lower levels of the taxonomy tree gives promising utility but privacy will be low. This results in a trade-off between privacy and utility which can be exploited by designing an adaptive data generalization mechanism where the identity vulnerability of each QI and diversity of sensitive attributes are integrated to reduce the privacy issues while improving the anonymous data utility. We obtain vulnerability and diversity values and use them in generalizing each QI values. A comprehensive overview of the proposed algorithm working in the form of the flow chart is presented in Figure 5.

Apart from the visual flow chart, a complete pseudo-code of the adaptive generalization is listed in Algorithm 2. Lines 1–3 implement the *D* and *E* calculations as well as the comparison with the relevant thresholds ($T_{d}$ and $T_{e}$). Lines 5–11 perform the lower level generalization for the highly diverse classes, and Lines 13–19 implement the higher-level generalization for low diverse classes. Lines 5–11 and 13–19 are the two blocks of if–else. The initial three steps in both blocks are identical, the main difference lies in Line number 8 and Line number 16, where higher level and lower level generalizations are performed respectively. Finally, anonymous data ($AD^{\prime }$) is returned by combining both classes of anonymity (Lines 22,23). The complete pseudo-code used to determine the generalization level adaptively for QI anonymization is presented in Algorithm 2.

**Algorithm 2:** Adaptive data generalization algorithm
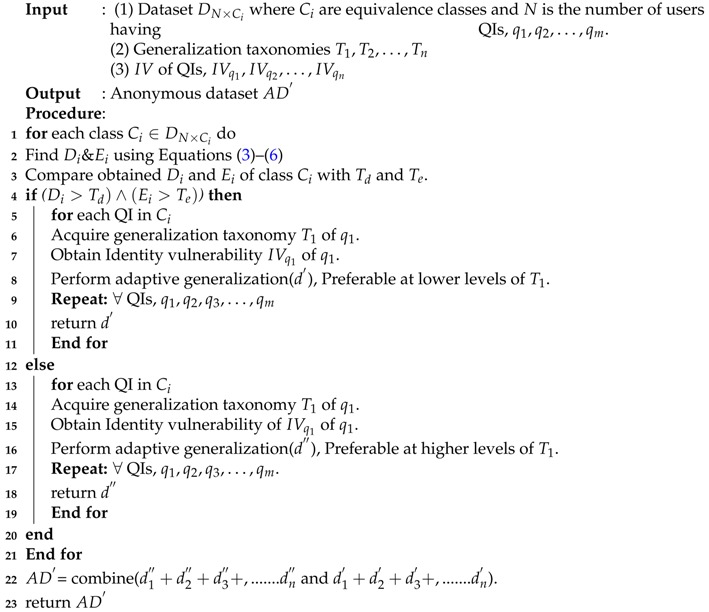


## 6. Simulation Results

This section demonstrates the output of the concepts discussed. The improvements of the proposed method are compared using three criteria—the improvements in user privacy, the anonymous data utility, and the computational overheads—with benchmark privacy-preserving algorithms. To benchmark the proposed method with other existing methods, the proposed method is compared with InfoGain Mondrian [41] and IACk [43], both of which have been demonstrated as being better than other methods in terms of utility and privacy when anonymizing data. Some readers may wonder why we compared our proposed algorithm results with Mondrian [41] and IACk [43] only. Basically, there are many similarities between our proposed algorithm and these two algorithms in terms of generalization level selection for anonymizing QI values, developing some criteria for data generalization, overcoming the information loss incurred from the generalization to improve anonymous data. Additionally, for building different data mining models, many adjustments for maintaining domain consistency in generalization process are required that are common among all three algorithms. Considering the identity vulnerabilities and diversities of the attributes result in significant improvements over these two algorithms results. That is why we compared our algorithm results with these two algorithms.

### 6.1. Dataset Description

The adult dataset originally extracted from census bureau database and currently found at U.C. Irvine Machine Learning Repository [51] has become a benchmark dataset for comparing *k*-anonymity algorithms. This dataset has been used by most of the *k*-anonymity studies (e.g., [52,53,54,55,56]).

The original dataset contains 48,842 instances, comprises of six numerical and eight categorical/non-numerical attributes and is 5.4 MB in size. Four attributes are used as QIs, and one attribute is used as the target class or SA. Other non-QI attributes from the dataset are ignored. There are several records in the dataset with missing values. The two-thirds division of actual data in the presence of missing values gives 32,561 instances as training data and 16,281 instances as testing data. We eliminated the records with unknown values before conducting experiments and resulting data set contains 45,222 tuples. The two-thirds division of refined data after eliminating missing values gives 30,162 instances as training data and 15,060 instances as testing data. A complete description of the dataset along with the generalization taxonomy levels used in the experiments is provided in Table 1.

In Table 1, the distinct values descend in the order of age (74), country (41), race (5), and gender (2). Salary is a sensitive attribute or target class with only two distinct values.

### 6.2. Improvements in Privacy

**Figure 6:**
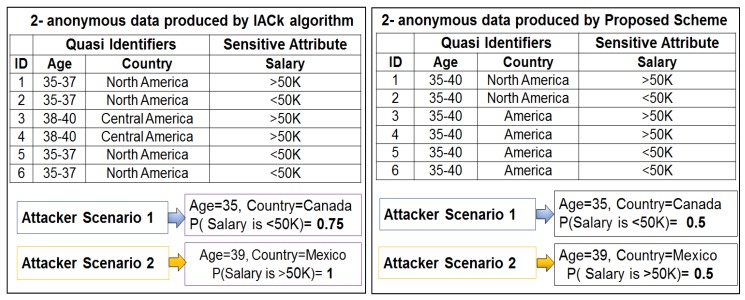
Comparison of privacy between the information-based anonymization for classification given *k* (IACk) algorithm and the proposed scheme.

The first set of analyses shows the improvements in user privacy provided by proposed scheme. The overall response to this criterion is good, and the anonymous data produced by the scheme is more resistant toward many attacks as compared to existing work. Identity revelation is reduced via the adaptive generalization, which considers the diversity of classes and identity vulnerability of each QI. The table shown in Figure 1a is used to highlight the major differences between existing studies and the present work. In this small subset of data, the age attribute potential was determined for identity revelation as compared to the country attribute because most of the tuples belong to the same territory. Age and country have IV values of 0.063 and 0.027, respectively. These findings confirm that age needs considerable attention during anonymization. Three classes—*A*, *B*, and *C*—are considered, as mentioned in Figure 1b. Determining class diversity is equally important because it limits private information disclosure. The Simpson index is employed to determine class diversity. Class *A* has a diversity of 2, which is higher than the threshold, but *B* and *C* do not have diversity. This indicates that age is highly capable of revealing identities, which is helpful information when anonymizing data. This study prefers higher level generalization rather than suppression to maintain data utility. One might argue that higher level generalization adversely affects utility, but at the same time, a lower level generalization of QIs with lower identity vulnerability and highly diverse classes will cancel this effect. Hence, there is no drastic change in data utility. Anonymous data produced by the scheme using the original data from Figure 1a is presented in Figure 6.

Six records are selected to show the major differences of the anonymization scheme with existing work. Adaptive data generalization ensures better protection for QIs with high identity vulnerability and less diverse classes. If the attacker already knows an individual‘s QIs, the scheme is still better at preserving privacy than the previous work. Two attacker scenarios with the possession of some QIs are also depicted in Figure 6. The proposed scheme on average achieves 37% better privacy protection regarding the linking attack. In Figure 6 symbol *P* represents the probability of re-identifying someone from an anonymous data. The probability of re-identification is close to the threshold (e.g., 1/k) as standard *k*-anonymity model. This ensures high-level protection even in class *A* for the QI that can potentially reveal an individual’s identity—i.e., age. Class *A* has sufficient diversity, and country is less identity vulnerable. Therefore, its values are retained as original (i.e., lower level generalization). In contrast, higher level generalization is recommended for classes *B* and *C* because they are not diverse and leak private information explicitly. The privacy improvements of the proposed scheme were compared with the IACk algorithm. Usually, the privacy analysis is based on auxiliary information (e.g., the information an adversary can get from the external sources). Therefore, our scheme made 37% improvements in the presence of auxiliary information with the standard deviation (*s*) of 0.11 and mean 0.5 as compared to IACk algorithm which has standard deviation (*s*) and mean ($\bar{x}$) values of 0.17 and 0.87 respectively.

### 6.3. Improvements in Anonymous Data Utility

During the anonymization of any data, information losses are inevitable. When data is distorted, some specific values are always lost. Meanwhile, many data values must be maintained in as original form as possible to enhance anonymous data utility. However, this can only be achieved when the data owner is fully aware of the data statistics (i.e., QIs having less identity vulnerability and classes with sufficient diversity). Therefore, an identity vulnerability- and diversity-aware anonymization scheme is needed to provide such valuable statistics to the data owner. By using these statistics for each attribute, over-generalizations can be controlled to reduce information losses, and the anonymous data better preserves the utility. To test utility, two criteria are used—information loss and classification model accuracy. Information loss is calculated using two metrics—distortion measures and coverage of generalized values. Both metrics are calculated using Equations (11) and (12), as explained in Section 6.3.1. A new variable—IV—is introduced in the formulas to take into account the identity vulnerability of each QI. Classification accuracy is calculated using three machine learning methods with the help of Equation (14) from Section 6.3.2. Table 2 shows the identity vulnerability values of different QIs present in the adult dataset. These measures are calculated with RF with a series of experiments.

Race and country are less identity vulnerable because the value distributions of these two attributes are skewed. For example, most records have United States listed as the country value, and the remaining records list many other countries. Therefore, these low-frequency values are more likely to appear in small-sized classes, and one can remove these low-frequency values. This explains why the identity vulnerability of these two attributes is lower as compared to age and gender. In contrast, the distribution of the gender attribute is almost even. Hence its vulnerability value is higher than those of country and race. Age has the highest number of distinct values and the distribution is even for all distinct values other than country, which has only one value with almost 90% of the overall values. Therefore, age has the highest identity vulnerability. The following setting with RF is used in order to determine the identity vulnerability of the QIs:

Number of trees (ntree) =500, QIs used to split the tree node (mtry) =3, RF model = classification, variable importance = true, keep.forest = true, data = users - data, predictors = (age, gender, race, country) and target = salary.

A parameter settings are performed, and Algorithm 1 is applied to determine the accurate values of the identity vulnerability of the QIs.

#### 6.3.1. Reduction in Information Losses

Data analysis applications want lower information losses with anonymous data to ensure better data utility. However, information loss is an unfortunate and inevitable consequence of any anonymization scheme. In the present experiments, information losses that occur during anonymization are quantified using two metrics—the distortion measure (DM) and the coverage of generalized values. Distortion is defined as the height of the taxonomy from the original value to the updated value [57] and it can be obtained using Equation (11).

(11) $$DM=\sum_{n=1}^{N}\sum_{q=1}^{Q}\frac{L_{i}}{L_{t}}\times IV_{q}$$

where $L_{i}$ is the level in the generalization taxonomy for which data is generalized, and $L_{t}$ represents the total number of levels in a taxonomy of QI values. $IV_{q}$ is the identity vulnerability of a QI. All distortion values from each QI and tuple are summed for all users in the dataset.

Table 3 shows the distortion measures achieved with the experiments on the adult dataset. Anonymous data produced by the adaptive generalization have lower average information losses as compared to existing schemes. The distortion is computed using Equation (11) and the average measures for different values of *k* are obtained and presented in Table 3. The proposed algorithm has less distortion as compared to existing schemes which do not take into account the identity vulnerability of each QI. Moreover, most of the improvements achieved by our proposed scheme regarding utility are derived from the fact that diversity and vulnerability are considered simultaneously during data generalization.

The coverage of generalized values is the total number of descendant leaf nodes of generalized values in the taxonomy [57].

Let *T* be the generalization taxonomy of a QI attribute. The coverage of a generalized QI value $va^{*}$ , denoted by $coverage[va^{*}]$, is given by the number of the descendant leaf values of value $va^{*}$ in *T*. The base of the taxonomy *T* is denoted by base(T), is the number of leaf values in the taxonomy. For example, in Figure 4b, $base(T_{race})=4$ since there are four leaf nodes in the generalization taxonomy, and $coverage[va^{*}=colored]=3$ since individuals having race values black, Asian-Pac-Islander and Amer-Indian-Eskimo can be generalized to colored in the generalization taxonomy from top to bottom. The information caused by the complete tuple in coverage of generalized values can be calculated by using Equation (12).

(12) $$IL\left(t^{*}\right)=\sum_{q\in Q}\left(IL\left(t^{*},q_{1}\right)\times IV\left(q_{1}\right)\right)$$

where $IL(t^{*},q_{1})$is the information loss caused by the coverage of the generalized values of a single QI computed from Equation (13), *Q* is a set of QIs, and IV is the identity vulnerability of specific QI $q_{1}$. The information loss caused by the coverage of generalizing for each QI is calculated using Equation (13).

(13) $$IL\left(t^{*},q_{1}\right)=\sum_{q=1}^{Q}\frac{coverage[va^{*}]-1}{base(T_{q})-1}\;if\;base\left[T_{q}\right]>1$$

Table 4 shows the comparison of the coverage of generalized values of proposed scheme with existing schemes. The present scheme consistently produces better average results for most values of *k*.

#### 6.3.2. Improvements in Classification Accuracy

In this section, the classification accuracy values obtained by building classification models on the anonymous data produced by this scheme are presented and compared with those of other schemes. Three different methods—RF, classification trees (CT), and support vector machines (SVMs)—are used to test the effectiveness of the proposed scheme regarding classification accuracy. Classification accuracy is calculated using the three machine learning methods (i.e., CT, SVM, and RF) and Equation (14).

(14) $$Accuracy\left(Acc.\right)=\frac{Tp+Tn}{N}$$

where $T_{n}$ is the number of true negatives, $T_{p}$ is the number of true positives, and *N* is the total number of users in the dataset. For classification utility, generalization is not an issue. Only domain consistency is important, so by making use of the identity vulnerability of the QIs, domain consistency is maintained. The classification accuracy is compared with that of InfoGain Mondrian [41], a benchmark utility-aware anonymization algorithm, and IACk [43], an extension of Mondrian that preserves classification utility using mutual information.

The implementation of the three methods is obtained using an R-tool [58], Weka [59], Matlab and Salford Predictive Modeller [60]. These environments are chosen for implementation because they are well-known, highly effective tools for statistical computing. The test dataset is independent of the corresponding training datasets. Generalization levels are determined from only the training data and are validated from testing data.

We calculated and compared the mean and standard deviation (SD) of accuracy values obtained from three different methods. Using the classification tree method, the proposed scheme gives the mean accuracy value of 82.08, as compared to IACk and Mondrian algorithms that give mean accuracy values of 80.75 and 80.25, respectively. We got 1.11 as the value of the SD of our proposed algorithm as compared to the IACk algorithm with an SD value of 0.98 and that of the Mondrian algorithm with an SD value of 0.93. Using the support vector machines method, we got a mean accuracy value of 84.08 of our proposed algorithm as compared to IACk and Mondrian algorithms that give mean accuracy values of 77.75 and 73.6 each. Our proposed scheme gives SD value of 1.51, as compared to IACk and Mondrian algorithms that give SD values of 4.3 and 2.3 respectively. The proposed scheme gives less estimates of SD as compared to other algorithms. However, the main reason is the difference between mean accuracy and each individual accuracy value for different values of *k*. As most of the individual accuracy values are close to the mean of accuracy, therefore the difference between actual value and mean ($x-\bar{x}$) is not sufficiently large. Due to this reason, the SD value of the proposed scheme is lower than the other two algorithms. Apart from classification tree and support vector machines, we calculated and compared the classification accuracy measures using random forest method too. The proposed scheme gives mean accuracy value of 88, as compared to Mondrian algorithm that gives mean accuracy values of 76.6. Our proposed scheme produces improved results while comparing SD value that is 1.47 as compared to Mondrian algorithms that give a SD value of 1.97. These results have further strengthened our confidence in anonymous data utility while providing sufficient privacy guarantees.

**Figure 7:**
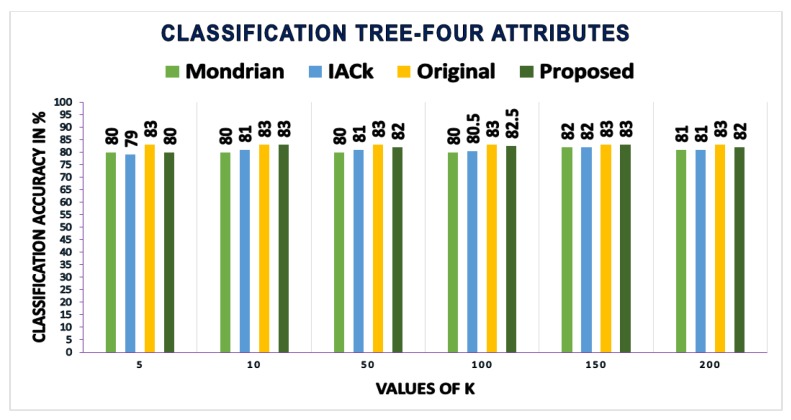
Accuracies: proposed scheme versus IACk and Mondrian algorithms.

**Figure 8:**
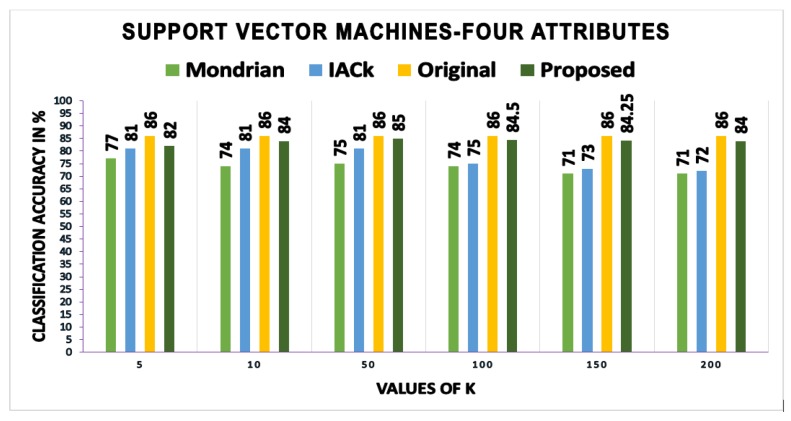
Accuracies: proposed scheme versus IACk and Mondrian algorithms.

**Figure 9:**
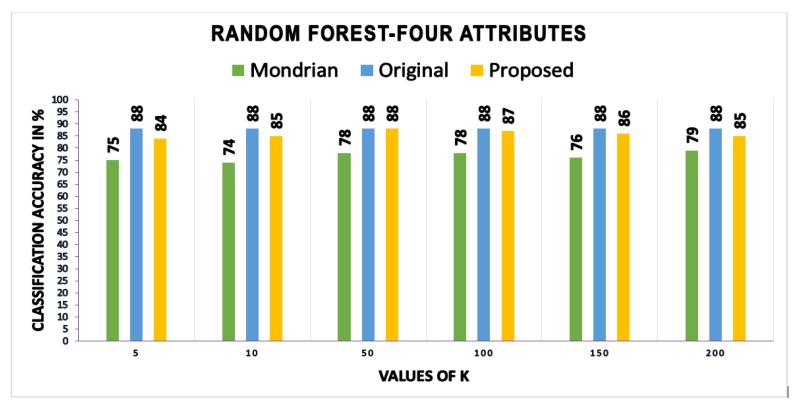
Accuracies: proposed scheme versus Mondrian algorithm.

Figure 7, Figure 8 and Figure 9 show the classification accuracy of the three different methods using four attributes on *k*-anonymized datasets with the proposed algorithm, InfoGain Mondrian [41] and IACk [43]. The proposed scheme also considers SA diversity in the anonymized datasets, which is often overlooked by classification-aware methods. Most classification-aware methods open the values that are suppressed by the methods discussed in the related work, while SA diversity, which makes private information disclosures obvious, is ignored. The accuracy of the models built on top of the anonymous dataset produced by our scheme is as good as the original data. The proposed algorithm is consistently better than those of Mondrian and IACk. The obtained accuracy values make the findings more reliable.

Generally, high accuracy of anonymous data is evaluated low in privacy in many real-world cases. Meanwhile, highly accurate data is evaluated low in privacy only when sensitive attribute diversity is not considered and considerable attention has not been paid to each QI identity revelation ability. However, the proposed scheme offers superior privacy protection considering the diversity and identity revelation ability of attributes and allows data users to classify two different income levels, such as, < or > than 50,000 dollars while using either some or all QIs as predictors.

We tested the algorithm on modified dataset containing a sensitive attribute in numbers and changing predictors; the RF can handle all types (e.g., numeric and categorical) of the predictor variable besides the type, the number of variables and data size. When the target/sensitive variable is numeric then the RF model will be "regression" rather than "classification" which is used for categorical variables. In adaptive data generalization, each attribute has domain generalization and corresponding value generalization taxonomy to create the anonymous version of original data. Therefore, data type will not affect the generalization process and data owners can create the data generalization taxonomies or can use the existing generalization taxonomies. The proposed scheme can work well with all types of the data.

### 6.4. Decrease in Computational Overheads

The identity vulnerability of the QIs is determined as an off-line task, and adaptive generalization is performed as an on-line task. Meanwhile, the running time of the proposed scheme sums both processes. Table 5 shows the execution time in seconds (s) and comparison of the present scheme with the InfoGain Mondrian scheme. The running time of proposed algorithm and Mondrian on the same data sets with different values of *k* were compared on a PC computer running windows operating system with CPU of 2.6 GHz and 8.00 GB RAM. When comparing the running times, the proposed anonymization scheme is faster.

These results emphasize the validity of the proposed scheme with respect to achieving better privacy protection, improved anonymous data utility, and low computational overheads. This study provides additional support for highly imbalanced data anonymization as compared to current state of the art methods. The findings appear to be well substantiated for both privacy and utility. The proposed scheme performs well regarding user privacy and anonymous data utility for two reasons: (1) the identity vulnerability of QIs is introduced, which helps to treat QIs according to their identity revelation ability and effectively protects user privacy; and (2) the adaptive generalization, which considers the identity vulnerability of the QIs and diversity of the SAs, improves the anonymous data utility by controlling over-generalization of diverse classes and less identity-vulnerable QIs. The proposed scheme resolves privacy issues stemming from low diverse classes and highly identity-vulnerable QIs, and it overcomes the difficulty of creating feasible *ℓ*-diverse datasets.

## 7. Conclusions

In this paper, we proposed a personally identifiable information (PII) vulnerability- and diversity-aware anonymization scheme to reduce the privacy risks while improving the anonymous data utility in the person-specific data publishing. The main goals of the proposed scheme are to reduce the unique identification and private information disclosures, and enhance anonymous data utility in the privacy preserving data publishing. We propose a mechanism for quantifying the identity vulnerability of each item of PII using random forest to reduce the unique identifications caused by the highly identity-vulnerable PII. We adapt Simpson index for calculating the diversity of equivalence classes to overcome the privacy breaches caused by low diverse classes. Furthermore, the proposed adaptive generalization scheme anonymizes data considering the identity vulnerability of PII as well as the diversity of equivalence classes. The adaptive generalization scheme resolves the privacy issues stemming from the highly identity-vulnerable PII and low diverse classes, and improves data utility by controlling the over-generalization of less identity-vulnerable PIIs. The proposed scheme results are promising with respect to privacy, utility, and computational overheads. Through simulations and comparison with the existing schemes, on average, our scheme reduces the privacy risks of identity and private information disclosures by 37%. From the anonymous data utility point of view, it lowers the information losses by 18%, and improves the classification models accuracy up to 6%.

## Figures and Tables

**Table 1:** Description of the adult dataset.

Attributes	Type	Quasi-identifier	Description
Age	Numerical	Yes	Taxonomy tree of height 7
Race	Categorical	Yes	Taxonomy tree of height 3
Gender	Categorical	Yes	Taxonomy tree of height 2
Country	Categorical	Yes	Taxonomy tree of height 4
Salary	Categorical	No	Sensitive Attribute

**Table 2:** Adult dataset: identity vulnerability values of QIs.

Sr.No	Quasi-Identifiers	Actual Values	Relative Values
1	Age	0.03	81.72
2	Gender	0.01	16.92
3	Race	0.00047	0.83
4	Country	0.00011	0.53

**Table 3:** Distortion measures (DM) comparison.

Values of *k*	Existing Schemes	Proposed Scheme
5	0.024	0.01
10	0.025	0.011
50	0.033	0.019
100	0.033	0.020
150	0.035	0.022
200	0.037	0.025
Mean	0.03	0.01
Standard Deviation	0.005	0.006

**Table 4:** Coverage of generalized values comparison.

Values of *k*	Existing Schemes	Proposed Scheme
5	8.3	2.5
10	12.3	3.7
50	16.3	4.9
100	33	9.9
150	68	52.5
200	160	60.5
Mean	49.6	22.3
Standard Deviation	58.32	26.70

**Table 5:** Running time (in s) of the proposed scheme versus Mondrian.

Values of *k*	Proposed Algorithm	Mondrian Algorithm
10	15 s	20 s
20	15 s	20 s
100	16 s	19 s
150	18 s	18 s
200	18 s	19 s
